# The Role of Cell Adhesion and Cytoskeleton Dynamics in the Pathogenesis of the Ehlers-Danlos Syndromes and Hypermobility Spectrum Disorders

**DOI:** 10.3389/fcell.2021.649082

**Published:** 2021-04-21

**Authors:** Sabeeha Malek, Darius V. Köster

**Affiliations:** Division of Biomedical Sciences, Centre for Mechanochemical Cell Biology, Warwick Medical School, University of Warwick, Coventry, United Kingdom

**Keywords:** Ehlers-Danlos syndrome, hypermobility spectrum disorder, fibroblasts, integrins, cytoskeleton, mechanobiology

## Abstract

The Ehlers-Danlos syndromes (EDS) are a group of 13 disorders, clinically defined through features of joint hypermobility, skin hyperextensibility, and tissue fragility. Most subtypes are caused by mutations in genes affecting the structure or processing of the extracellular matrix (ECM) protein collagen. The Hypermobility Spectrum Disorders (HSDs) are clinically indistinguishable disorders, but are considered to lack a genetic basis. The pathogenesis of all these disorders, however, remains poorly understood. Genotype-phenotype correlations are limited, and findings of aberrant collagen fibrils are inconsistent and associate poorly with the subtype and severity of the disorder. The defective ECM, however, also has consequences for cellular processes. EDS/HSD fibroblasts exhibit a dysfunctional phenotype including impairments in cell adhesion and cytoskeleton organization, though the pathological significance of this has remained unclear. Recent advances in our understanding of fibroblast mechanobiology suggest these changes may actually reflect features of a pathomechanism we herein define. This review departs from the traditional view of EDS/HSD, where pathogenesis is mediated by the structurally defective ECM. Instead, we propose EDS/HSD may be a disorder of membrane-bound collagen, and consider how aberrations in cell adhesion and cytoskeleton dynamics could drive the abnormal properties of the connective tissue, and be responsible for the pathogenesis of EDS/HSD.

## Introduction

The Ehlers-Danlos syndromes (EDS) are a group of heritable connective tissue disorders defined by the presence of three clinical features: joint hypermobility, skin hyperextensibility, and tissue fragility (Malfait et al., 2017). The condition is named after two dermatologists, Edvard Lauritz Ehlers and Henri-Alexandre Danlos both of whom independently characterized some of the first clinical descriptions of EDS in the early 20th Century (Parapia and Jackson, 2008). The formal categorization of the EDS subtypes began in the 1960s, and there have since been several reclassifications following advances in our understanding of the molecular and genetic basis of these disorders (Table 1). Most subtypes have now been shown to be caused by mutations in genes affecting the structure or processing of collagen (Malfait et al., 2017). Most recently, the International Consortium on the ehlers-danlos syndromes proposed updated diagnostic criteria in 2017, which recognizes 13 different EDS subtypes (Malfait et al., 2017). Since its publication, a 14^th^ subtype of EDS has been identified (Blackburn et al., 2018). While provisional diagnoses can be made based on clinical major and minor criteria, a definitive diagnosis now relies on the molecular identification of the causative variant(s) in the respective gene. The exception lies with the hypermobile form of EDS (hEDS; formerly EDS type III) which, despite being the most common form, remains as the only subtype without an identified distinct genetic variation, and whose diagnosis remains clinical alone. In addition, it is important to recognize the related Hypermobility Spectrum Disorders (HSD) which are clinically indistinguishable from hEDS, and both diagnoses are often termed together as hEDS/HSD. HSD is a diagnosis the EDS consortium had intended to reflect patients who demonstrate the characteristic feature of hEDS, i.e., joint hypermobility, but lack sufficient clinical evidence to demonstrate a genetic aetiology to their presentation. The segregation of these diagnoses was intended to produce a more homogenous cohort to further aid research efforts to identify the genetic markers of hEDS, but does not differentiate patients in terms of severity of symptoms or disability (Tinkle et al., 2009; Copetti et al., 2019). Collectively, EDS/HSD has a diagnosed prevalence of 1 in 500 (Demmler et al., 2019).

**TABLE 1:** Past and present classifications of EDS subtypes and their molecular basis.

2017 International Classification of EDS (2017-present) (Malfait et al., 2017)	Villefranche criteria (1998–2016) (Beighton et al., 1998)	Berlin Nosology (1988–1998) (Beighton et al., 1988)	Genetic basis	Protein	OMIM condition
Classical EDS (cEDS)	EDS Classical Type	Gravis (EDS type I)	COL5A1	Type V collagen	130000
		Mitis (EDS type II)	COL5A2		130010
	**	**	COL1A1	Type I collagen	–
Classical-like EDS (clEDS) type 1	**	**	TNXB	Tenascin XB	606408
**Classical-like EDS type 2	**	**	AEBP1	Aortic Aarboxypeptidase-Like Protein	618000
Cardiac-valvular (cvEDS)	**	**	COL1A2	Type I collagen	225320
Vascular EDS (vEDS)	EDS Vascular Type	Arterial-ecchymotic (EDS type IV)	COL3A1	Type III collagen	130050
	**	**	COL1A1	Type I collagen	–
Hypermobile EDS (hEDS)	EDS Hypermobility Type	Hypermobile (EDS type III)	Unknown	Unknown	130020
Arthrochalasia (aEDS)	EDS Arthrochalasia Type	Arthrochalasis multiplex congenita (EDS type VIIA)	COL1A1	Type I collagen	130060
	**	Arthrochalasis multiplex congenita (EDS type VIIB)	COL1A2	Type I collagen	617821
Dermatosparaxis EDS (dEDS)	EDS Dermatosparaxis Type	Human Dermatosparaxis (EDS type VIIC)	ADAMTS2	Procollagen I N-proteinase	225410
Kyphoscoliotic EDS (kEDS)	EDS Kyphoscoliosis Type	Ocular-Scoliotic (EDS type VIA)	PLOD1	Lysyl hydroxylase 1	225400
	**	**	FKBP14	FK506 Binding Protein 22kDa	614557
Brittle Cornea syndrome (BCS)	**	Ocular-Scoliotic (EDS type VIB)	ZNF469	Zinc finger protein 469	229200
	**	**	PRDM5	PR domain-containing protein 5	614170
Spondylodysplastic EDS (spEDS)	Other forms (Progeroid EDS)	**	B4GALT7	β-1,4-galactosyltransferase 7	130070
	**	**	B3GALT6	β3GalT6	615349
	**	**	SLC39A13	ZIP13	612350
Musculocontractural EDS (mcEDS)	**	**	CHST14	Dermatan-4 sulfotransferase-1	601776
	**	**	DSE	Dermatan sulfate epimerase-1	615539
Myopathic EDS (mEDS)	**	**	COL12A1	Type XII collagen	616471
Periodontal EDS (pEDS)	Other forms (Periodontal type)	Periodontitis type (EDS type VIII)	C1R	C1r	130080
			C1S	C1s	617174
(X-linked cardiac valvular dysplasia)*	Other forms (X-linked EDS)	X-linked type (EDS type V)	FLNA	Filamin-A	314400
Occipital horn syndrome (OHS)*	Occipital horn syndrome (OHS)*	X-linked cutis laxa (EDS type IX)	ATP7A	ATPase, Cu (2++)-transporting, alpha polypeptide	304150
*	Other forms (Fibronectin-deficient EDS)	Fibronectin-deficient (EDS type X)	–	–	225310
Familial hypermobility syndrome (FHS)*	Other forms (Familial hypermobility syndrome)	Familial articular hypermobility syndrome (EDS type XI)	–	–	147900

*Removed as an EDS subtype from the criteria.**Unidentified or unrecognized EDS subtype at time of criteria formation.

**FIGURE 1:**
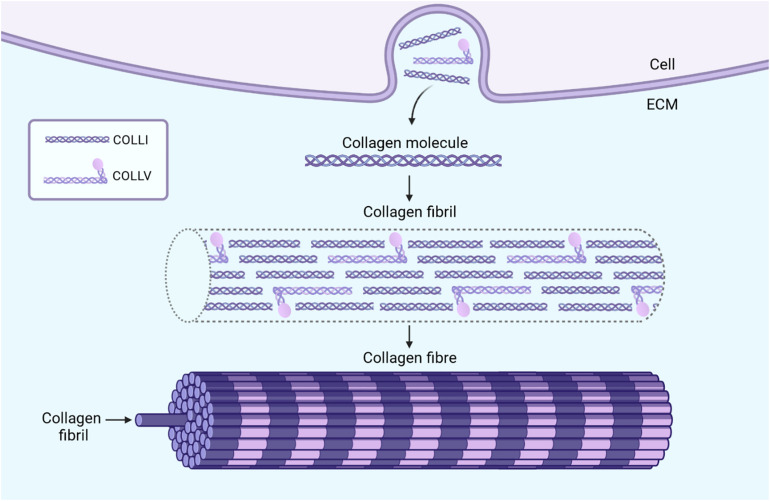
The formation and function of collagen fibrils in connective tissue. Fibroblasts secrete collagen molecules into the ECM which associate in a staggered pattern to form collagen fibrils. These collagen fibrils consist mostly of the major collagen protein, type I, while the minor collagen proteins, such as type III and type V constitute only a fraction of fibril mass (Theocharidis and Connelly, 2019). The importance of these minor collagens, however, lies in their role in regulating the diameter and organization of collagen fibrils. In the example of type V collagen molecules, the presence of a non-collagenous domain which projects outwards introduces steric hindrances when incorporated within collagen fibrils (Wenstrup et al., 2004a). This limits the lateral growth of the fibrils and may also play a role in regulating their diameter (Wenstrup et al., 2004a). Multiple collagen fibrils together form collagen fibers which provide tensile strength to the connective tissue. The absence of sufficient amounts of minor collagen proteins, like in EDS, can result in poorly formed collagen fibrils, and in turn, collagen fibers (Theocharidis and Connelly, 2019). The tissue specific expression and roles of the minor collagen proteins presumably accounts for the characteristic presentation of each specific EDS subtype, all of which differ in the varying presence, manifestation, and degrees of joint hypermobility, skin hyperextensibility, and tissue fragility. *Image created with BioRender.com*.

Our current understanding of EDS/HSD pathogenesis is informed by the two best characterized subtypes, the classical (cEDS) and vascular (vEDS) forms, which are consequent to mutations in genes encoding the minor collagen proteins, type V (Symoens et al., 2012), and type III (Pepin et al., 2000), respectively. Mutations either cause haploinsufficiency, which is a 50% reduction in protein expression caused by a nonsense-mediated decay of the non-viable RNA transcript (Leistritz et al., 2011; Symoens et al., 2012), or the production of a structurally defective procollagen molecule which is retained within the endoplasmic reticulum (Smith et al., 1997; Symoens et al., 2012). Consequently, in both scenarios, the absence of sufficient functional minor collagen proteins in the extracellular matrix (ECM) then impedes with their role in regulating the formation and organization of the major type I collagen into fibrils (Figure 1; Liu et al., 1997; Wenstrup et al., 2004a,b; D’hondt et al., 2018; Wang et al., 2020). This disrupts the integrity of the ECM and is assumed to be the underlying cause of connective tissue weakness.

However, this notion is not truly reflected in transmission electron microscopy (TEM) analyses of collagen fibril structure and organization in EDS/HSD skin biopsies, though general abnormalities can be found. These abnormalities include reduced and disorganized collagen bundles with abnormal orientations, or non-circular cross-sections of collagen fibrils that are described as “flower-like,” or with variable diameters both larger and smaller than would be typically expected (Holbrook and Byers, 1982; Hausser and Anton-Lamprecht, 1994; Hermanns-Lê and Piérard, 2006). vEDS patients have been characterized as having collagen fibrils that are smaller and more variable, and with an overall reduced density (Smith et al., 1997), while cEDS is associated with the presence of flower-like collagen fibrils (Vogel et al., 1979; de Moraes et al., 2000; Bowen et al., 2017; Angwin et al., 2020).

Yet, these findings are not consistent and this should be considered significant. It has long been noted that the degree of ultrastructural changes observed do not correlate with clinical severity (Proske et al., 2006), or presentation (Kobayasi, 2004; Hermanns-Lê et al., 2012). Further studies have shown that patients can present with no significant collagen abnormalities, despite a clinical presentation and/or confirmation of genetic disorder. In 2019, one study reported two individuals with a likely pathogenic variant of the COL5A1 gene that did not present with the typical collagen flowers expected (Angwin et al., 2019), contradicting a consensus previously reached by the EDS committee in 2017 (Bowen et al., 2017). Another study in vEDS patients found that the size of collagen fibers and bundle characteristics did not discriminate between vEDS and control participants, nor did all vEDS participants present with abnormal fibril diameters (Ong et al., 2012). More recently, a larger study of 177 EDS patients found that no specific TEM finding could be associated with any specific EDS subtype (Angwin et al., 2020). Remarkably, from the 177 patients with a clinical diagnosis, 147 (83%) had a normal TEM report, and from the 24 patients with a genetic confirmation of their subtype, 7 (29%) also had a normal biopsy report (Angwin et al., 2020). Conversely, it has been demonstrated that clinically unaffected family members and control participants can also present with the same EDS-associated ultrastructural abnormalities without displaying features of a connective tissue disorder (Kobayasi, 2004; Hermanns-Lê et al., 2012). As such, the only conclusion that can be derived from these collective TEM studies is that an abnormal biopsy finding is more frequently found in EDS/HSD patients (Angwin et al., 2020).

These findings demonstrate that despite the categorization of EDS/HSD as a disorder of collagen, the reported ultrastructural collagen abnormalities do not appear to be the principal mediators of abnormal connective tissue properties in these disorders. It is therefore plausible to assume that other related factors heavily influence connective tissue properties and mediate the EDS/HSD phenotype. One such factor may be the cellular mechanics of fibroblasts, the predominant cell type that populates the connective tissue.

## The Role of Fibroblasts in Mediating the Properties of the Connective Tissue

**FIGURE 2:**
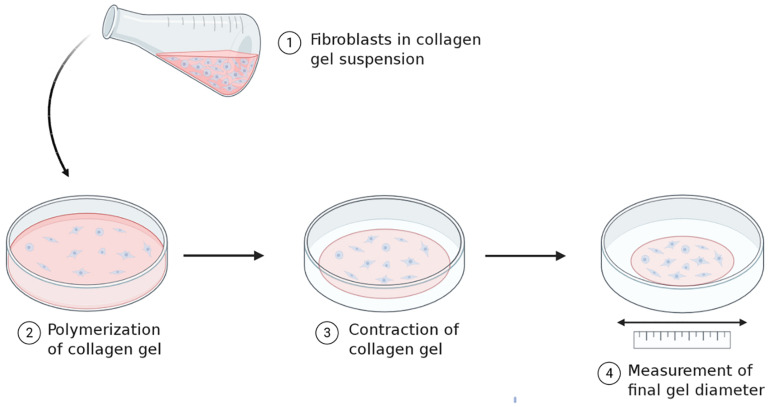
Principles underlying collagen gel contraction assays. Standard gel contraction assays involve seeding fibroblasts into a collagen gel solution and allowing the suspension to polymerize. The freshly polymerized gel is placed in media for a set time period and allowed to contract. Changes in the diameter of the gel are measured before and after and can be used as a parameter to quantify cell contractility. *Image created with BioRender.com.*

The ability of fibroblasts to directly exert force and influence the tension in the surrounding environment is significant, as it directly affects the viscoelastic properties of the connective tissue. This has been demonstrated through gel contraction assays (Figure 2) (Dallon and Ehrlich, 2008). Fibroblasts seeded into isolated and recombinant collagen gels generate tractional forces by cytoskeleton contractility which propagate throughout the gel matrix. This compacts the collagen fibrils, eliminating water from between the fibrils which decreases the gel volume. The contraction of this gel is proposed to reflect the ability of fibroblasts to contract an open wound, hence this is used as the typical model assay for the study of cell-ECM interactions in the context of wound healing.

Such gel contraction assays have provided several relevant findings of note. Firstly, the ability of fibroblasts to contract the gel significantly, demonstrates how the cellular mechanics of fibroblasts can directly influence the material properties of their environment, e.g. the connective tissue (Delvoye et al., 1991; Eastwood et al., 1994, 1996). These assays have also demonstrated the key cellular features enabling gel contraction. One is cell adhesion to collagen via integrins, as blocking integrin-mediated adhesion with antibodies prevented gel contraction (Klein et al., 1991; Schiro et al., 1991; Kelynack et al., 2000). Equally important is an active cytoskeleton, as using molecular inhibitors that interfere with actin filament and microtubule polymerization abrogates tractional force generation and reduces tension within the gel (Kolodney and Wysolmerski, 1992; Swierczewski et al., 2016; Giannopoulos et al., 2018). The involvement of intermediate filaments has also been indicated in this process, as vimentin-null fibroblasts show a reduced ability to contract the gel (Ridge et al., 2016).

However, the significance of these findings is not just/limited to the context of wound healing, but reflect important processes involved in the ongoing maintenance of connective tissue function. It has been shown that fibroblasts do not simply contract the gel when seeded, but they alter their cytoskeletal contractility to adapt to any external loads applied in order to maintain an overall resting tension in the environment (Delvoye et al., 1991; Brown et al., 1998; Mizutani et al., 2004; Giannopoulos et al., 2018; Zollinger et al., 2018). Indeed, the specific level of resting tension established by fibroblasts has been described as a “tensile setpoint”, and their tendency to actively maintain this equilibrium as “tensional homeostasis” (Brown et al., 1998). Furthermore, it has been shown that the ability to maintain tensional homeostasis is not dependent on the stiffness of the environment, and is only limited by the load force not the displacement (Freyman et al., 2002). This suggests that healthy fibroblasts are also capable of compensating for a materially weak ECM by adjusting the level of contractile force needed to maintain overall normal levels of resting tension (Tomasek et al., 2002). Experiments on mouse subcutaneous connective tissue have also reproduce these findings in a more physiological setting. Stretching connective tissue samples caused a disproportionate change in fibroblast morphology, which could not be explained by simple passive spreading of cells, but indicated an active response (Langevin et al., 2005, 2011). The importance of active processes in mediating tensional homeostasis was shown when inducing cell death or inhibiting cytoskeleton dynamics increased the tension within the connective tissue by 60-80% upon stretch (Langevin et al., 2011).

These findings indicate that fibroblast mechanics play a significant role in mediating the properties of the connective tissue beyond the context of wound healing. Fibroblasts actively alter their contractile forces in response to everyday mechanical strains and loads for the purposes of maintaining appropriate tension, hence, also determining the viscoelastic properties of the connective tissue. Furthermore, these processes are dependent on the key processes of cell adhesion and cytoskeleton dynamics. Impairments in any of these processes, therefore, may affect connective tissue integrity and viscoelasticity, and contribute to the pathogenesis of EDS/HSD.

Indeed, fibroblast dysfunction has been implicated in the pathogenesis of several other connective tissue disorders. Fibroblasts from patients with floppy eyelid syndrome, a hyperelasticity disorder affecting the upper eyelid, demonstrate a significantly higher tensile setpoint compared to control (Ezra et al., 2010). In Dupuytren’s contracture, a condition affecting the facia of the hand, fibroblasts are unable to respond appropriately to mechanical loading, and exert an opposite response and of a greater magnitude compared to control fibroblasts, something which also disturbed the attainment of tensional homeostasis (Bisson et al., 2004, 2009). Of particular significance are findings from Pelvic Organ Prolapse (POP) patients, a condition of weakened connective tissue highly associated with EDS/HSD (Carley and Schaffer, 2000; Lammers et al., 2012). Under static conditions, POP fibroblasts in cell culture demonstrate a significantly higher expression of the cytoskeleton proteins actin, α-tubulin, and vimentin compared to control cells, indicating an increased mechano-response to the stiff substrate of a culture dish (Wang et al., 2015). In response to mechanical strain, however, POP fibroblasts exhibit a significant decrease in the expression of actin, in contrast to control cells that increased actin expression under the same conditions (Wang et al., 2015). Another study also found that POP fibroblasts delayed the alignment of their actin cytoskeleton in the direction of the force in response to mechanical strain compared to control cells (Ruiz-Zapata et al., 2013). These findings indicate that POP fibroblasts are unable to efficiently respond to mechanical forces, which may even overload and impair the integrity of cytoskeletal system. In the context of a whole tissue environment, such a failure would contribute to the weakening of the connective tissue, and similar mechanisms may exist and contribute to the pathogenesis of EDS/HSD.

## EDS/HSD Fibroblasts Exhibit Relevant Integrin-Mediated Changes in Cell Adhesion and Cytoskeleton Organization

**FIGURE 3:**
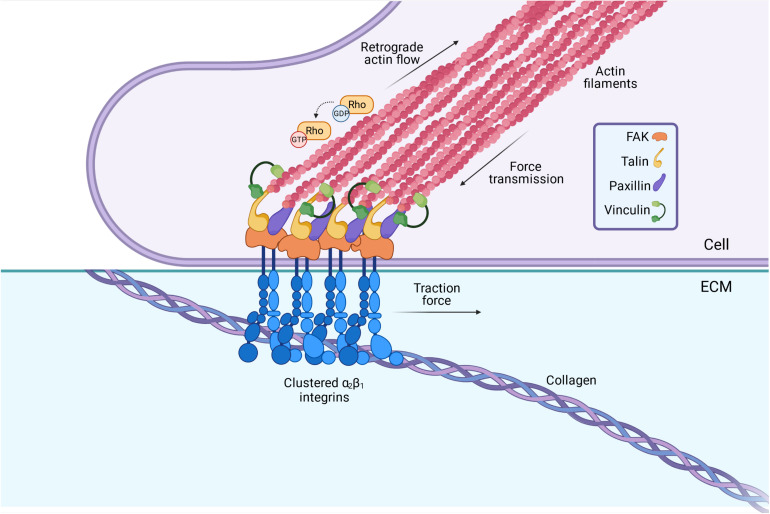
Integrins serve as a vital connection between the cells’ internal cytoskeleton and the ECM. Integrins span the membrane of fibroblasts, with vital intracellular and extracellular domains. Binding of the ECM ligand (collagen, fibronectin etc.) to the extracellular domain leads to the activation of the integrin, and a conformational change in its cytoplasmic tail. This triggers a rapid recruitment of adaptor proteins such as talin and paxillin to the cytoplasmic tail, which in turn, triggers a signaling cascade involving the Rho GTPases. These act as molecular switches to regulate the polymerization of actin, and hence, play an important role in determining the cytoskeletons contractility. The lateral assembly or “clustering” of integrins also occurs upon ligand binding, to form multi-protein complexes termed “focal adhesions” at the cell surface. These act as strong anchoring points and mediate the specific attachment between the cell cytoskeleton and the ECM. *Image created with BioRender.com.*

The culturing of fibroblasts derived from the dermal biopsies of EDS/HSD patients have revealed several relevant molecular changes (Zoppi et al., 2018b; Chiarelli et al., 2019), including an altered integrin expression profile that is shared amongst the main EDS/HSD subtypes (Zoppi et al., 2018b). Integrins are cell surface receptors composed of one α and one β subunit, giving rise to 24 unique integrins which play a central role in cell adhesion, cell signaling, and cell survival (Barczyk et al., 2010). Integrins mediate the attachment between the cell and specific ECM proteins like collagen or fibronectin (Figure 3). Binding of the ECM ligand exposes the cytoplasmic tail of the integrin, which provides a scaffold to allow recruitment of paxilin, vinculin, talin, and other proteins during the formation of multi-protein complexes called focal adhesions constituting signaling complexes (Kanchanawong et al., 2010; Parsons et al., 2010). These in turn, regulate the activity of the Rho family of GTPases, which act as molecular switches to regulate downstream signaling processes and cytoskeleton organization (Kanchanawong et al., 2010; Parsons et al., 2010). These adhesion sites act as a strong anchoring point for the actin cytoskeleton, important for the mechanical feedback between cell and ECM.

The integrin profile demonstrated in EDS/HSD involves the downregulation of the collagen receptor, α_2_β_1_ integrin, as well as the fibronectin receptor, α_5_β_1_ integrin, while the vitronectin receptor, α_v_β_3_ integrin, is upregulated instead. In EDS/HSD, this α_v_β_3_ integrin functions as an alternative receptor for fibronectin (Zoppi et al., 2018b). The expression of this specific integrin profile is termed the “integrin switch” (Zoppi et al., 2004, 2018b), which can be recreated in control cells with a functional blocking of the α_2_β_1_ receptor (Zoppi et al., 2004). This demonstrates that the absence of a structurally organized collagen in the ECM failing to engage with its receptor α_2_β_1_ leads to the altered integrin expression profile. This, in turn, will influence other cellular processes that may be critical in the pathogenesis EDS/HSD.

The first of these is mechano-sensitivity, which is an ability of fibroblasts to sense the compliance of their environment and the strain present within it (Schwartz, 2010). This process would be essential to produce the appropriate cytoskeletal response to mechanical strain and requires the cooperative actions of various integrins. A study demonstrating such cooperation of integrins with different mechanosensitive roles showed the α_5_β_1_ integrins to be implicated in force generation, whereas α_v_-class integrins mediated the structural adaptations to forces (Schiller et al., 2013). Fibroblasts in which the interaction between the α_v_β_3_ integrin and fibronectin is blocked, also failed to sense the rigidity of a fibronectin matrix (Jiang et al., 2006). This would suggest that the integrin switch would alter the mechano-sensing ability of EDS/HSD fibroblasts. Indeed, there is already some indication that this process is perturbed in hEDS/HSD. When hEDS/HSD fibroblasts were cultured on a stiff culture substrate, excessive actin stress fibers could be observed compared to control (Zoppi et al., 2018a), however, these were absent in hEDS/HSD fibroblasts that were directly observed within tissue samples (Celli et al., 2020). This demonstrates that excessive stress fiber formation is not a persistent feature of hEDS/HSD fibroblasts, but instead, could be reflective of mechano-sensitivity aberrations. It is possible that this abnormal cytoskeletal response only occurred when fibroblasts adhered to stiff substrates *in vitro*, but not when they remained within their native soft tissue environment. Such mechano-sensitivity aberrations are physiologically relevant, as conditions of mechanical strain can temporarily stiffen the environment of fibroblasts *in vivo*, and, similarly, produce an abnormal cytoskeletal response which contributes to abnormal connective tissue properties.

The integrin switch may also impair cell-ECM adhesion, which may be crucial for tissue integrity. Indeed, aberrant adhesion has already been demonstrated in EDS/HSD. In cEDS and vEDS fibroblasts, cell-ECM adhesion has been shown to be mediated specifically between the α_v_β_3_ integrin and fibronectin, whereas control cells show no such critical dependency (Zoppi et al., 2008). The fibronectin organization itself is impaired in EDS/HSD, present only as rare fibrils in the ECM (Zoppi et al., 2018b), further indicating a compromised cell-ECM interaction. The strength of the cell-ECM adhesions themselves, may also be limited. A single bond between the α_2_β_1_ integrin and collagen can withstand a mechanical force of 160 pN (Niland et al., 2011), whereas bonds between α_5_β_1_ and fibronectin begin to break at 30 pN (Kong et al., 2009). Bonds between α_v_β_3_ and fibronectin break at even lower forces (Roca-Cusachs et al., 2009). This was demonstrated in a study where adhesion between cells and fibronectin-coated beads was mediated either by the α_v_β_3_ integrin or α_5_β_1_. Following application of a 1 nN force for 100 s, cells adhering via α_v_β_3_ completely detached from the beads, whereas cells adhering via the α_5_β_1_ integrin maintained adhesion (Roca-Cusachs et al., 2009). The same differences in adhesion strength were also observed when clustering of the integrins was promoted (Roca-Cusachs et al., 2009). It is suggested that these weaker α_v_β_3_ bonds facilitate force detection by breaking more easily, and hence, their role is to enable mechano-transduction rather than adhesion. Consequently, these collective findings suggest that the integrin switch, alongside the defective fibronectin organization observed in EDS/HSD, may produce far weaker connections between the cell and ECM. These may break more easily under conditions of mechanical strain, and contribute to the weakening of the integrity of the connective tissue (Roca-Cusachs et al., 2009).

Some impairments in the cytoskeleton of EDS/HSD fibroblasts have also been observed *in vitro*. cEDS and vEDS fibroblasts demonstrate a disorganized actin cytoskeleton (Zoppi et al., 2008), whereas in hEDS and HSD, the actin cytoskeleton is organized into stress fibers (Zoppi et al., 2018a). Furthermore, cEDS and vEDS fibroblasts show a decreased migratory capacity (Viglio et al., 2008; Zoppi et al., 2018a), whereas cell migration is significantly increased in hEDS and HSD fibroblasts (Zoppi et al., 2018a). This migratory capacity is relevant, as it involves the same mechanical machinery that generates the tractional forces implicated in mediating tensional homeostasis (Pollard and Borisy, 2003; Webster et al., 2014). These findings indicate that this aspect of EDS/HSD fibroblast function may also be aberrant and contribute to abnormal connective tissue properties.

The integrin switch itself may also have further implications for cytoskeleton dynamics. Integrins are also known to influence the activities of the Rho-GTPases, RhoA, Rac1, and Cdc42, which are crucial to cell protrusion formation (Lawson and Burridge, 2014), which in turn, is involved in the tensional homeostasis mechanism (Webster et al., 2014). During this process, the initial extension of the plasma membrane is driven predominantly through Rac-mediated actin polymerization, while RhoA and Cdc42 activity contributes to extension of cell protrusions at later stages of cell spreading (Ridley, 2015). It has been demonstrated that β1 integrin subunit in particular, is required to support RhoA activation at later stages of cell spreading (Danen et al., 2002). Overexpression of the β3 subunit has also been shown to enhance the activity of RhoA and promote stress fiber formation, whereas overexpression of the β1 subunit enhanced Rac activity and cell protrusion formation (Miao et al., 2002). In another study examining cell migration in epithelial cells, adhesion via α_v_β_*3*_ was shown to support extensive actin cytoskeletal reorganization and the formation of a single broad protrusion at the leading edge, whereas cell adhesion via α_*5*_β_*1*_ caused the extension of thinner protrusions (Danen et al., 2005). These findings indicate that the integrin profile demonstrated in EDS/HSD cells, may also have direct consequences on the ability of fibroblasts to form and/or maintain cell protrusions under conditions of mechanical strain and maintain connective tissue tension.

## Assessing Dermatosparaxis and EDS Fibroblast Dysfunction Through Gel Contraction Assays

It is clear from the molecular changes described that fibroblast dysfunction is to be expected in EDS/HSD, involving aberrations in both cell adhesion and cytoskeleton dynamics. This has been directly examined in gel contraction studies performed with fibroblasts from animals with dermatosparaxis, which is equivalent to the human form of dermatosparaxis EDS (dEDS; formerly EDS type VIIC) (Colige et al., 1999). This disorder is caused by mutations in the ADAMTS2 gene, which encodes the procollagen N-proteinase enzyme, and consequently results in poorly structured and loosely arranged collagen fibrils in the ECM (Bavinton et al., 1985). However, similarly, to the ongoing discrepancies in human EDS/HSD studies, the TEM findings of aberrant collagen fibrils in dermatosparaxis animals do not appear to reflect severity of presentation, and therefore, fail to fully account for and explain the abnormal properties of the connective tissue (Bavinton et al., 1985). The gel contraction abilities, however, do more accurately reflect clinical presentation. Dermal fibroblasts from mildly affected sheep demonstrate a gel contraction profile approaching that of control dermal fibroblasts (Ramshaw et al., 1991), while those from severely affected calf and sheep failed to contract the gel (Delvoye et al., 1983, 1986, 1991; Ramshaw et al., 1991). Furthermore, fibroblasts obtained from the less affected tissues in the severely presenting calf, like the tendons, vena cava, and aorta, also reflect this tissue specific presentation, and contracted the gel, similarly, to control fibroblasts (Delvoye et al., 1983). These finding are highly significant, as the correlation of fibroblast dysfunction with severity of manifestation, both between differently presenting animals, and between tissues within the same animal, likely demonstrates one of the main pathomechanisms mediating the observed connective tissue abnormalities. This provides confirmation that dysfunction of the fibroblast does indeed, constitute a relevant pathomechanism for EDS/HSD, and one that has now been demonstrated in a relevant animal model.

The nature of the fibroblasts inability to contract the gel was later shown to be due to the cell surface absence of a 34 kDa collagen binding protein related to anchorin CII (Mauch et al., 1988), which prevented effective cell adhesion of dermatosparatic fibroblasts to collagens type I and IV (Mauch et al., 1986). Consequently, it appears these fibroblasts were unable to attach to the type I collagen in the gel and effectively mediate the cytoskeletal forces needed to contract the collagen gel, as well as maintain homeostatic levels of tension within a restrained gel (Delvoye et al., 1991). These findings, therefore, confirm that impaired cell adhesion is a significant feature of fibroblast dysfunction, reducing integrity of the connective tissue integrity.

These findings highlight the complexity that underlies monogenic disorders. Pathogenesis of dermatosparaxis in this case, may not have been principally mediated by the aberrant collagen molecule itself, but mediated by a reduced cell surface expression of a cell adhesion molecule, that initially appears unrelated to the underlying ADAMTS2 mutation. This demonstrates that far more complex cellular and molecular processes underlie the pathway from initial mutated gene to final pathogenic phenotype and is likely to involve the interplay of various independent environmental and related factors. These independent factors could influence critical pathogenic features, and either promote a less or more severe phenotype in the affected individuals, which may help to explain the vast heterogeneity seen in the presentation of these disorders.

The same gel contraction studies were also performed with human EDS samples at the time, however, they demonstrated unexpected results. Fibroblasts from patients of several EDS subtypes showed no gel contraction abnormalities and behaved similar to control fibroblasts (Delvoye et al., 1986, 1991). Remarkably, this also included dEDS, which has an identical genetic basis to dermatosparaxis. It was concluded from these studies that fibroblast dysfunction did not contribute to the pathogenesis of human EDS/HSD, and research into this pathomechanism discontinued as focus turned toward identifying further EDS-related genes.

However, this conclusion now appears contradictory to our molecular understanding of EDS/HSD. The α_2_β_1_ integrin is the main collagen receptor in humans and plays the equivalent role to the anchorin CII-related protein in mediating the attachment between the cell and collagen. The α_2_β_1_ integrin is also downregulated in EDS/HSD fibroblasts as part of the integrin switch (Zoppi et al., 2018b). Furthermore, several gel contraction studies performed in control cells while blocking the collagen-α_2_β_1_ interaction, prevented contraction of the gel (Klein et al., 1991; Schiro et al., 1991; Kelynack et al., 2000). These findings all highly indicate that the integrin switch seen in EDS/HSD fibroblasts should result in similar impairments in cell-ECM adhesion and prevent gel contraction, while also reflecting their pathological behavior *in vivo*.

Our understanding of the mechanisms regulating these molecular processes has also progressed. It is now apparent that these gel contraction assays do not truly reflect the EDS/HSD scenario in humans specifically, and hence, the drawn conclusions may not be valid. It has since been demonstrated that adding relevant collagen to the media of cultured human EDS fibroblasts promotes phenotypic correction, which reverses the integrin switch and restores the expression of α_2_β_1_ to the cell surface (Zoppi et al., 2004). As such, the collagen supplied in these gel contraction assays may have corrected the phenotype of human EDS fibroblasts, and restored the cell surface expression of α_2_β_1_. This would have promoted normal gel contraction, yet not reflect the true *in vivo* behavior of EDS/HSD fibroblasts. Hence, the human equivalent of this pathomechanism cannot be demonstrated through standard gel contraction assays. The existence of this pathomechanism therefore, has not been excluded in human patients, nor has it yet been truly examined.

## Discussion

**FIGURE 4:**
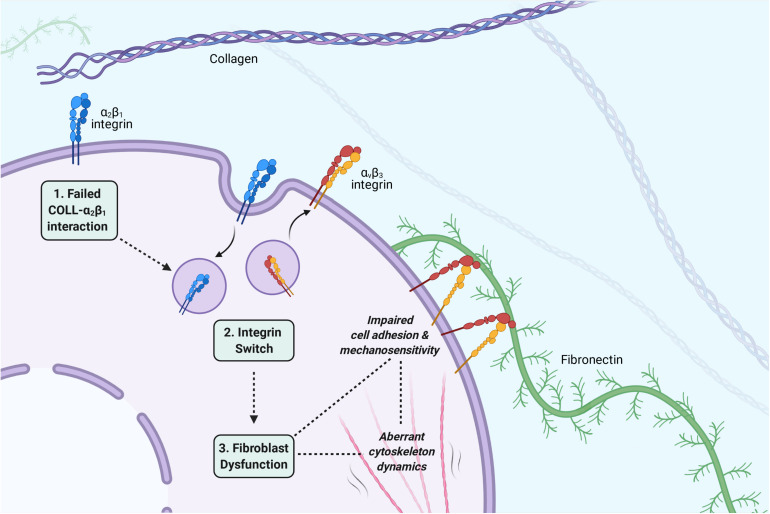
The three principal stages of the proposed pathomechanism for EDS/HSD. (1) A failed interaction between collagen and α_2_β_1_: A disorganized or defective collagen-ECM prevents engagement of the collagen ligand with its receptor, the α_2_β_1_ integrin, and triggers the integrin switch. (2) The integrin switch: Fibroblasts respond to the defective collagen-ECM by switching their cell adhesion profile to promote adhesion to other ECM ligands. The α_2_β_1_ and α_5_β_1_ integrins are downregulated, and α_v_β_3_ is upregulated, which then mediates the attachment between cell and ECM via fibronectin. (3) Fibroblast dysfunction: The altered integrin profile also has various consequences for the fibroblasts phenotype, which affects its ability to implement the tensional homeostasis mechanism and maintain integrity of the connective tissue. Impaired cell-ECM adhesion may promote fragility of the connective tissue. Impairments in mechanosensitivity may cause an incorrect interpretation of tension within the connective tissue and facilitate an abnormal cytoskeleton response. Aberrations in the dynamic cytoskeleton response itself, may also facilitate abnormalities in the viscoelastic properties of the connective tissue. All such aspects may contribute to the EDS/HSD phenotype. *Image created with BioRender.com*.

This review had the aim to highlight a potential pathomechanism in EDS/HSD involving fibroblast dysfunction. Three principal stages of this pathomechanism can be described (Figure 4).

The first is a failed interaction between collagen and its receptor, the α_2_β_1_ integrin (Zoppi et al., 2018b). The underlying cause of this failed interaction is specific for each EDS subtype, and can easily be accounted for in the genetically characterized subtypes of EDS. In cEDS and vEDS for example, mutations in the minor collagen genes prevent the organization of type I collagen into fibrils, which prevents its engagement with α_2_β_1_ (Zoppi et al., 2018b). In hEDS and HSD, though the principle underlying molecular defect is unknown, an enhanced expression of the matrix metalloproteinase (MMP), MMP-9, has been observed (Zoppi et al., 2018a). The proteolytic activity of MMP-9 could promote a general disassembly of the ECM, and in turn, prevent the engagement of collagen with α_2_β_1_. This stage of the pathomechanism, however, also allows the possibility of an acquired EDS phenotype, consequent to any other disease processes that interferes with collagen-α_2_β_1_ binding. Speculatively, any autoimmune or inflammatory condition that promotes a general disassembly of collagen in the ECM could initiate the same pathomechanism and promote connective tissue abnormalities, which then manifest through features of joint hypermobility, skin hyperextensibility, and tissue fragility.

The second stage of the pathomechanism involves the integrin switch itself, which is a shared feature of the main EDS/HSD subtypes (Zoppi et al., 2018b). Following the failed collagen-α_2_β_1_ interaction, a downregulation of the α_2_β_1_ integrin itself occurs, which is accompanied by a downregulation of the fibronectin receptor α_5_β_1_, and an upregulation of the alternative fibronectin receptor α_v_β_3_. It is possible that fibroblasts are able to sense the compromised collagen-ECM via the α_2_β_1_ integrin, and recognize a potential risk of *anoikis*, a specific form of cell death that occurs upon cell detachment from the ECM. To avoid such a fate, it is possible that fibroblasts respond by switching their integrin profile to promote cell adhesion to other ECM ligands, such as fibronectin, in order to maintain some form of attachment to the ECM and survive. Indeed, this notion is supported by apoptosis assays performed in cEDS and vEDS fibroblasts, where the α_v_β_3_ integrin was specifically shown to rescue EDS cells from *anoikis* (Zoppi et al., 2008), indicating that this integrin switch is indeed, a critical cell survival response. The importance of cell-ECM adhesion, however, extends beyond the transmission of cell survival signals, and we have described in this review its importance in maintaining connective tissue integrity. The integrin switch, therefore, may form a key stage of this pathomechanism, due to the consistent nature of this finding across all of the main EDS/HSD subtypes, as well as the critical consequences in weakening cell-ECM adhesion and its contribution to overall fibroblast dysfunction.

The final stage of this pathomechanism is fibroblast dysfunction itself, which ultimately mediates the abnormal properties of the connective tissue. The critical features of this dysfunction are impairments in cell adhesion and cytoskeleton dynamics. As already described, impaired cell-ECM adhesion weakens the integrity of the connective tissue, however, it also prevents fibroblasts from mediating their cytoskeletal forces to the surrounding environment and implement the tensional homeostasis mechanism. The integrin switch itself, has also been shown to directly affect cytoskeleton dynamics, impairing the cytoskeletal forces that are transferred through the weakened cell-ECM adhesions. The integrin switch may also cause abnormalities in the mechanosensitivity of fibroblasts, which may prevent accurate interpretations of the surrounding ECM properties, and hence, may produce an inappropriate response by fibroblasts. As such, key connective tissue properties such as viscoelasticity and integrity are all affected consequent the integrin switch and may contribute to the pathogenesis of EDS/HSD.

The contribution of this specific pathomechanism to the overall EDS/HSD phenotype could potentially be significant. It has to be highlighted that the current TEM analyses of collagen fibril structure and organization in EDS/HSD are highly inconsistent, and fail to associate with any EDS subtype, specific connective tissue abnormality, or severity of the manifestation (Angwin et al., 2020). Indeed, even the most significant abnormality associated with EDS/HSD, in the form of collagen flowers in cEDS, is one that cannot explain the disproportionate changes in skin viscoelasticity seen in this subtype, and is also one that is found in unaffected individuals (Hermanns-Lê et al., 2012). To our knowledge, the studies into fibroblast mechanics in dermatosparaxis animals, are the only non-genetic findings to have correlated with severity of connective tissue manifestation (Delvoye et al., 1983; Ramshaw et al., 1991), yet the possible significance of this appears to have been overlooked.

These findings suggest that what unites and defines the EDS/HSD subtypes may not be defects in the structure and/or organization of collagen itself, but in cell adhesion to collagen. If such a notion is correct, this may provide an ideal basis for the development of a universal or non-specific diagnostic test that truly captures all EDS/HSD subtypes, irrespective of the principal underlying defect. An assessment for membrane-bound collagen for example, would highly indicate that cell-ECM adhesion has switched *in vivo* from collagen to other ECM ligands, reflecting the occurrence of the initial stages of this pathomechanism in patients presenting with connective tissue ailments. This is of importance, since the most common forms of hEDS and HSD, are currently without any diagnostic biomarker, and it is likely that further unidentified subtypes remain. This may also have implications for other related disorders such as fibromyalgia, and Myalgic Encephalomyelitis/Chronic Fatigue Syndrome (ME/CFS), which demonstrate significant overlaps with hypermobility and connective tissue abnormalities, though the association is poorly understood (Bragée et al., 2020; Eccles et al., 2020). It is plausible therefore, that similar pathomechanisms involving fibroblast dysfunction may be contributing to the pathogenesis of these related disorders, which could be explored through their associations with membrane-bound collagen.

Of additional importance is to determine if this pathomechanism contributes to arterial fragility in vEDS, a lethal subtype of EDS with a median life expectancy of 40 years (Eagleton, 2016). Death commonly occurs due to complications associated with spontaneous vascular or hollow organ ruptures, however, the development of these are also poorly understood (Eagleton, 2016). If such a pathomechanism demonstrates itself to be significant, the development of a therapeutic agent that promotes arterial integrity via these processes could provide hope for a number of patients.

## Author Contributions

SM and DK conceived this manuscript. SM wrote the initial draft of the manuscript. DK reviewed, added, and edited the content of the manuscript. Both authors contributed to and approved the final manuscript for submission.

## Conflict of Interest

The authors declare that the research was conducted in the absence of any commercial or financial relationships that could be construed as a potential conflict of interest.
